# A giant mystery in giant cell myocarditis: navigating diagnosis, immunosuppression, and mechanical circulatory support

**DOI:** 10.1002/ehf2.12564

**Published:** 2019-12-24

**Authors:** Julianne M. Fallon, Alex M. Parker, Steven P. Dunn, Jamie L.W. Kennedy

**Affiliations:** ^1^ Department of Pharmacy Cleveland Clinic Cleveland OH; ^2^ Division of Cardiology University of Florida Health Gainesville FL USA; ^3^ Department of Pharmacy University of Virginia Health System Charlottesville VA USA; ^4^ Division of Cardiology University of California San Francisco San Francisco CA USA

**Keywords:** Giant cell myocarditis, mechanical circulatory support, immunosuppression

## Abstract

Giant cell myocarditis is a rare but often devastating diagnosis. Advances in cardiac imaging and mechanical circulatory support have led to earlier and more frequent diagnoses and successful management. This disease state has wide variation in acuity of presentation, and consequently, optimal treatment ranging from intensity and type of immunosuppression to mechanical circulatory support is not well defined. The following case describes the management of a patient with an unusual presentation of giant cell myocarditis over a 10 year course of advanced heart failure therapies and immunomodulatory support. This case highlights emerging concepts in the management of giant cell myocarditis including sub‐acute presentations, challenges in diagnosis, and treatment modalities in the modern era.

## 1 Introduction

Giant cell myocarditis (GCM) is a rare, incompletely understood disease. Severe, rapidly progressive heart failure in young to middle‐aged adults characterizes the most frequently reported presentation of GCM.^1^ Prognostic differences have been suggested on the basis of initial presentation of myocarditis, with patients in fulminant myocarditis and histologically proven GCM both highly associated with short‐term mortality.^2^ Early reports of GCM demonstrated survival rates around 5 1/2 months after diagnosis; however, modern reports have shown 5 year survival free from death or heart transplant of 52–72% with immunosuppression.^3^, ^4^, ^5^ Some patients respond to immunosuppression; many require transplantation for long‐term survival.^4^ Increased use of mechanical circulatory support (MCS) over the past decade has played a pivotal role in improving survival and time to transplant. The optimal balance between immunosuppression, MCS, and heart transplant has not been fully elucidated in GCM. The following case describes an atypical presentation of GCM managed with a combination of immunosuppression, left ventricular assist device (LVAD), and eventually orthotopic heart transplantation (OHT).

## 2 Case report

A 54‐year‐old Caucasian woman presented with symptoms of chest tightness, palpitations, and non‐sustained ventricular tachycardia (VT). Her past medical history was significant for supraventricular tachycardia; she took no prescription medications and lived in an area with high prevalence of tick‐borne illnesses. Cardiovascular family history included heart failure (mother) and supraventricular tachycardia (daughter). Her physical examination was notable for clear lungs and no jugular venous distension, gallops, or murmurs. Vitals included a heart rate of 68 beats per minute, blood pressure of 100/60 mmHg, height of 64 inches, and weight of 62.5 kg. Her admission electrocardiogram showed biventricular bigeminy and Q waves in leads V1 and V2. Her labs were normal except for B‐type natriuretic peptide of 237 pg/mL (reference range < 100 pg/mL) and troponin I 0.16 ng/mL (reference range < 0.08 ng/mL). Transthoracic echocardiogram demonstrated normal biventricular function, mild mitral regurgitation, mild pulmonary hypertension, and an estimated left ventricular ejection fraction (LVEF) of 60%. Coronary angiography revealed no coronary disease, and she was discharged on metoprolol, lisinopril, and mexiletine.

**Figure 1:**
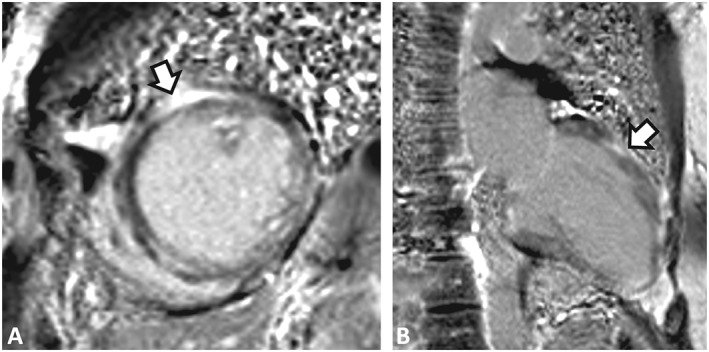
Cardiac magnetic resonance imaging. (A) Mid short axis late gadolinium enhancement (LGE) image. (B) Two‐chamber LGE image. There is a distinctive pattern of diffusely located “patchy” transmural delayed hyper‐enhancement compatible with infiltrative cardiomyopathy and fibrosis of the left ventricle. This pattern is not indicative of ischaemic cardiomyopathy. There is subepicardial LGE along the mid‐anterior wall, which can be seen in myocarditis.

She was admitted three times the following year for VT with persistently elevated troponin levels (0.12–0.42 ng/mL). Her LVEF had declined to 30–35%, and a dual chamber implantable cardioverter‐defibrillator was implanted. Cardiac magnetic resonance imaging found subepicardial hyper‐enhancement along the mid‐anterior wall of the left ventricle compatible with myocarditis and LVEF of 32.8% (*Figure* 1). Her metoprolol was increased on discharge.

**Figure 2:**
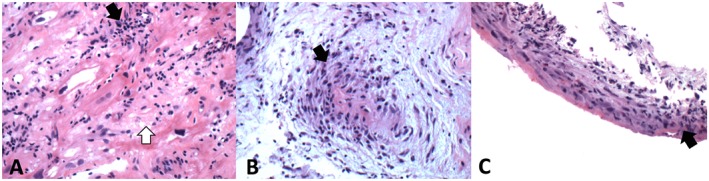
Endomyocardial biopsy. (A) High‐power hematoxyylin and eosin (H&E) stained image showing markedly damaged myocardium with intermyocyte fibrosis and a diffuse infiltrate of lymphocytes. (B) High‐power H&E stained image showing a capillary surrounded with lymphocytic infiltrate. (C) Low power H&E stained image showing endocardium with a predominantly lymphocytic infiltrate. Black arrows indicate lymphocytic infiltrate and white arrows indicate damaged myocardium.

**Figure 3:**
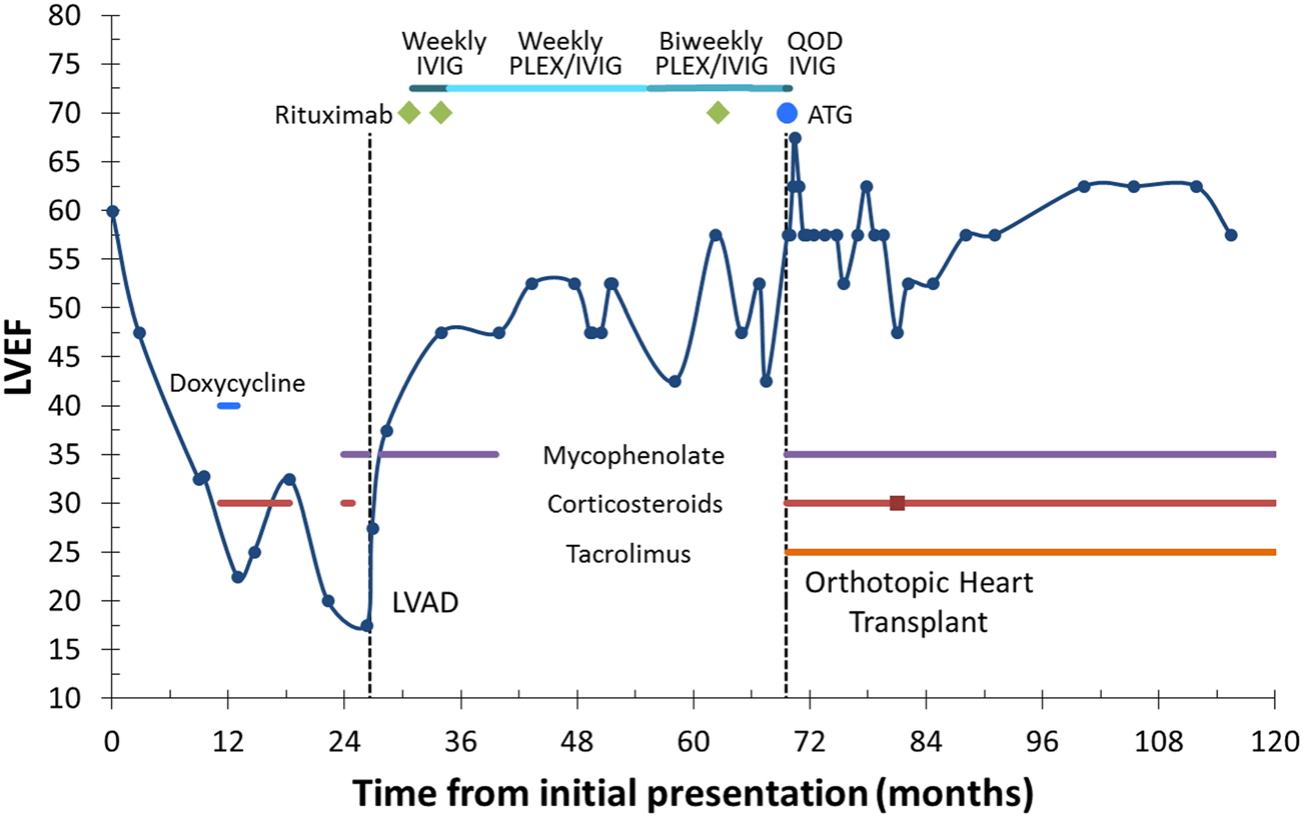
Timeline of case report showing LVEF over the 10 year follow‐up, treatment regimens, and time of LVAD and OHT relative to initial presentation. ATG, anti‐thymocyte globulin; IVIG, intravenous immunoglobulin; LVAD, left ventricular assist device; LVEF, left ventricular ejection fraction; PLEX, plasmapheresis; QOD, every other day.

She was referred to an academic medical center for endomyocardial biopsy, which showed inflammatory findings thought to be most consistent with Lyme carditis, staining for amyloid was unrevealing (*Figure* 2). Serologies for Lyme disease were negative. Treatment consisted of doxycycline, corticosteroid taper, and mycophenolate mofetil. A depiction of pharmacological therapy received during the patient's course is summarized in Figure 3. She had gradual improvement in LVEF that correlated with corticosteroid treatment and declined when corticosteroids were stopped. Despite treatment, her clinical status declined with recurrent heart failure admissions requiring inotropes, inability to tolerate evidence‐based medical therapy, worsening LVEF, and recurrent VT, leading to consideration of advanced heart failure therapies.

**Figure 4:**
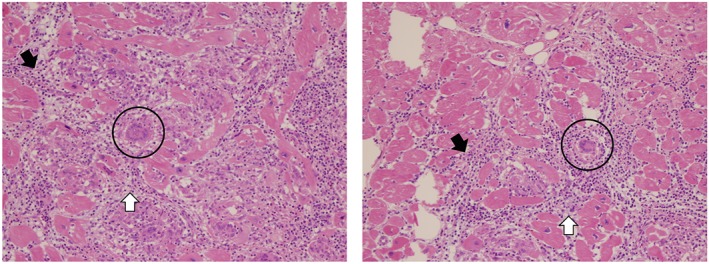
Left ventricular apex core biopsy. Both views are high‐power hematoxyylin and eosin (H&E) stained images showing markedly damaged myocytes with a lymphocytic infiltrate with the addition of multinucleated giant cells, not associated with granulomas. Black arrows indicate lymphocytic infiltrate, white arrows represent necrotic myocardium, and giant cells are circled in black.

Two years after initial presentation, a HeartMate II LVAD (Thoratec, Pleasanton, CA) was implanted as a bridge to transplant. Left ventricular apex core biopsy demonstrated a focal chronic lymphocytic infiltrate notable for multiple multinucleated cells and absence of granulomas, consistent with GCM (Figure 4). Additionally, staining for mycobacteria, atypical mycobacteria, bacteria, and fungi was negative. After LVAD implantation, she restarted mycophenolate mofetil for approximately 1 year, transitioned to azathioprine for a few days, and then antimetabolites were withdrawn because of leukopenia. Time to transplant was prolonged because of the presence of high levels of preformed antibodies. She received antibody reduction therapy with rituximab and intravenous immunoglobulin 4 months following LVAD implant; plasma exchange was added 4 months later because of persistently high levels of preformed antibodies. During this time, she experienced an improvement in symptoms and left ventricular function; however, she was unable to tolerate a trial wean of LVAD support to 8400 rpm, with a VO_2_ max of 10 mL/kg/min and peak respiratory exchange ratio of 1.3. More aggressive immunosuppression regimens targeting GCM were ultimately not pursued because of infection risk.

A suitable donor with negative prospective crossmatch became available, and she underwent an OHT at age 60. Her induction immunosuppression included three doses of rabbit anti‐thymocyte globulin, methylprednisolone 1 g followed by 250 mg every 8 h for six doses, and mycophenolate 1 g intraoperatively followed by 1 g twice/day. Maintenance immunosuppression included tacrolimus, prednisone, and mycophenolate. She is now 4 years post‐transplant, doing well without cellular or antibody‐mediated rejection or evidence of recurrent GCM.

## 3 Discussion

Classic reports of GCM include patients with acute onset heart failure rapidly progressing to cardiogenic shock and death within weeks to months of initial presentation.^4^, ^6^, ^7^, ^8^ GCM is often associated with refractory ventricular arrhythmias or other conduction abnormalities.^4^, ^7^, ^9^, ^10^ Only 14–29% of patients initially present with VT compared with more than 75% of patients presenting with acutely decompensated heart failure.^1^ These figures are likely a reflection of historical findings when GCM was only identified at the time of autopsy. A change in the acuity of patients diagnosed with GCM may be seen with increasing utilization of prospective endomyocardial biopsy and apex biopsy at the time of LVAD implantation. This sub‐acute case managed with immunosuppression and LVAD, followed by OHT with no GCM recurrence after 4 years, likely represents an example of possible benefit of early GCM diagnosis.

Diagnosis of GCM is often challenging because of the focal nature of the disease and similarities with other infiltrative cardiomyopathies; this is demonstrated in our patient who had definitive evidence of GCM only after left ventricular apex biopsy. Prospective endomyocardial biopsy has been the gold standard for the diagnosis of GCM since the 1980s because of the similarity in clinical features with other etiologies of myocarditis, particularly sarcoidosis, with the caveat that repeat biopsies are often necessary.^1^, ^5^ Targeted biopsies based on the findings of cardiac magnetic resonance imaging and other imaging studies may lead to increased diagnosistic accuracy of initial endomyocardial biopsy. The pathologic diagnosis of GCM requires exclusion of other causes of myocarditis including infectious diseases, amyloidosis, and sarcoidosis. GCM pathophysiology is linked to a T‐cell‐mediated process, characterized by infiltration of cardiomyocytes by lymphocytes, histocytes, multinucleated giant cells, eosinophils with necrosis of cardiomyocytes, and notable absence of granulomas.^1^, ^4^, ^11^, ^12^, ^13^

Saltykow first described GCM in 1905, and the role of immunosuppression was reported in 1987.^14^, ^15^ There are no prospective clinical trials to guide management of this rare disease; however, retrospective reports and case series suggest maintenance immunosuppressive therapy with or without induction. Induction regimens may include high dose steroids, muromonab CD3, or ATG, while maintenance therapy typically includes a corticosteroid, antimetabolite, and calcineurin inhibitor.^4^, ^16^ Survival beyond 1 year after diagnosis of GCM is rare without treatment; however, use of immunosuppression has been associated with extended survival.^3^, ^4^ Choosing an optimal evidence‐based immunosuppression regimen is challenging because of overall paucity of evidence because of the small number of reports and absence of data from randomized clinical trials. On the other hand, descriptions of successful use of MCS in patients with GCM have followed the increasing use of this modality in heart failure.^17^, ^18^, ^19^ Antibody reduction therapy with mycophenolate, rituximab, intravenous immunoglobulin, and plasma exchange coincided with improvement in our patient's symptoms and cardiac function, which offers an area for future study as T cells are traditionally thought to be the main GCM etiology.

Goals of GCM treatment have been to control symptoms, prolong life, and delay transplant. Heart transplantation is not necessarily a curative treatment, with approximately 20–25% of patients experiencing recurrence.^3^, ^20^ There may be a prognostic difference between symptomatic and asymptomatic biopsy proven recurrence and consequently management of each may vary.^17^ Fortunately, our patient has not had recurrence to date. Contemporary reports on survival in GCM have described survival after OHT at 1, 5, and 10 years as similar to other forms of myocarditis (94%, 82%, and 68%; *P* = 0.11). 21 Our patient highlights an example of an uncomplicated post‐transplant course after GCM diagnosis.

## 4 Conclusions

Advances in diagnostic modalities and treatment options are leading to earlier diagnosis and improved outcomes in GCM. Rare diseases are challenging to study with prospective clinical trials and clinicians often seek retrospective reports for guidance; registry data have filled this void in other uncommon conditions and may be beneficial in GCM. This case demonstrates a slowly progressive course, requiring durable MCS 2 years after presentation at which time the diagnosis of GCM was made. Intriguingly, cardiac function improved during antibody reduction therapy, though MCS could not be weaned and OHT was ultimately pursued. This suggests an area for further research into alternative methods to treat GCM. This case provides considerations for the utilization of synergistic imaging and treatment modalities for patients with GCM in order to promote early identification and ultimately reduce symptoms and improve functional status.

## Conflict of interest

None declared.
